# Effect of *Wenshen-Yanggan* Decoction on Movement Disorder and Substantia Nigra Dopaminergic Neurons in Mice with Chronic Parkinson's Disease

**DOI:** 10.1155/2020/9838295

**Published:** 2020-06-21

**Authors:** Lili Tang, Chang Chen, Baomei Xia, Wei Wu, Ruide Wei, Guoxue Zhu, Juanjuan Tang, Xin Zhou, Yan Liang, Zhen-Nian Zhang, Yan Lu, Ye Yang, Yang Zhao

**Affiliations:** ^1^Nanjing University of Chinese Medicine, Nanjing, Jiangsu, China; ^2^Department of Neurology, Nanjing Hospital of Chinese Medicine Affiliated to Nanjing University of Chinese Medicine, Nanjing, Jiangsu, China; ^3^Medical School of Nanjing University, Nanjing, Jiangsu, China; ^4^Faculty of Rehabilitation Science, Nanjing Normal University of Special Education, Nanjing, Jiangsu, China; ^5^School of Medicine and Life Sciences, Nanjing University of Chinese Medicine, Nanjing, Jiangsu, China; ^6^Center for Modernization of Chinese Medicine and Database, Nanjing Hospital of Chinese Medicine Affiliated to Nanjing University of Chinese Medicine, Nanjing, Jiangsu, China; ^7^School of Basic Biomedical Science, Nanjing University of Chinese Medicine, Nanjing, Jiangsu, China

## Abstract

This study aimed to explore the protective effects of *Wenshen-Yanggan* decoction on dopaminergic (DA) neuron injury in a rotenone-induced mouse model with chronic Parkinson's disease (PD) and explore its mechanism of action. Ultraperformance liquid chromatography-tandem mass spectrometry (UPLC-MS/MS) was used to measure the content of six main components in the *Wenshen-Yanggan* decoction. The chronic PD mouse model was established by treating 10-month-old healthy wild C57BL/6 male mice with rotenone 30 mg/kg/day for 28 days in succession. The pole test and rotarod test were applied to detect the rescue effect of *Wenshen-Yanggan* decoction in high, medium, and low dosages, respectively, on PD-like behaviors in mice with chronic PD. The protective effect of *Wenshen-Yanggan* decoction on the mesencephalic nigrostriatal DA neuron injury was determined employing tyrosine hydroxylase (TH) immunofluorescence staining. Enzyme-linked immunosorbent assay (ELISA) was adopted to measure the inflammatory cytokines in serum, including TNF-*α* (tumor necrosis factor-alpha), IFN-*γ* (interferon gamma), NF-*κ*B (nuclear factor kappa-B), and IL-1*β* (interleukin-1 beta). Western blotting was performed to quantify the expression of phosphorylated *c*-Jun N-terminal kinase (*p*-JNK), cleaved caspase-3, B-cell lymphoma-2 (Bcl-2), and NF-*κ*B in the brain. Our results showed that the *Wenshen-Yanggan* decoction in high, medium, and low dosages reduced the turning time of mice (*P* < 0.01, *P* < 0.01,  and *P* < 0.05). The high and medium dosages shortened the total climbing time of PD mice in the pole test (*P* < 0.01 and *P* < 0.05). Meanwhile, the high, medium, and low dosages increased the rod-standing time of PD mice in the rotarod test (*P* < 0.01, *P* < 0.05,  and *P* < 0.05). Besides, the decoction reversed the decrease in TH-positive neurons induced by rotenone, upregulated TH protein expression, and downregulated the *α*-syn expression in the PD model. Moreover, the decoction in high dosage significantly inhibited the expression of *p*-JNK, cleaved caspase-3, and NF-*κ*B in the midbrain of PD mice (*P* < 0.05, *P* < 0.05,  and *P* < 0.01), upregulated the expression of Bcl-2 (*P* < 0.05), and decreased the content of TNF-*α*, IFN-*γ*, NF-*κ*B, and IL-1*β* in the serum (*P* < 0.001, *P* < 0.001, *P* < 0.001,  and *P* < 0.001). Taken together, the *Wenshen-Yanggan* decoction could protect mice from rotenone-induced chronic PD, which might be related to the reduction of the DA neuron apoptosis via suppressing the inflammatory reaction and the neuronal apoptosis pathway.

## 1. Introduction

Parkinson's disease (PD) is the second most common age-related neurodegenerative disease. It is characterized by progressive loss of dopamine (DA) neurons mainly in the substantia nigra pars compacta (SNpc) and the appearance of cytoplasmic inclusion, which is called Lewy bodies, and its main component is *α*-synuclein. The autopsy analysis of PD patients and experimental animals indicates that proinflammatory factors and nigral dopaminergic neuron apoptosis were significantly increased, the common characteristics of the PD brain [1–3]. Previous pathology studies in PD patients and animal models and the neuroinflammation target treatment studies all indicated that the neuroinflammation and apoptosis response can mediate PD-related progressive death of dopaminergic neurons, suggesting inflammation and apoptosis could be considered as a promising target for PD treatment [4, 5].

Rotenone (RT) is a mitochondrial complex I inhibitor that can selectively impair dopaminergic neurons and induce PD-like behaviors [6]. This toxin features high lipophilicity and can easily access dopaminergic neurons beyond the blood-brain barrier without any transporter [7]. It is one of the neurotoxic drugs used to establish the neurotoxin model of PD in recent studies [8]. The rotenone model summarizes the majority of pathological features observed in PD patients, including the absence of dopaminergic neurons in the substantia nigra pars compacta and the enhanced oxidative stress and neuroinflammation in the nigrostriatal dopaminergic pathway [9].

Levodopa is the most effective drug for PD treatment at present; however, it cannot postpone the disease progression. Moreover, after levodopa intervention for 3–5 years, nearly all patients with PD develop motion complications such as reduced efficacy, on-off phenomenon, and dyskinesia. In traditional Chinese medicine, PD is called “tremor palsy,” which is characterized by tremors, numbness, and weakness of limbs. Accumulating literatures have reported the application of herbal prescriptions in alleviating PD symptoms, and the efficacy of Chinese herbal medicines has attracted increasing attentions. The *Wenshen-Yanggan* decoction is a formula used for PD treatment at Nanjing TCM Hospital, which is composed of Cistanches Herba, Paeoniae Radix Alba, Dioscoreae Rhizoma, Linderae Radix, Fructus Alpiniae Oxyphyllae, and Uncariae Ramulus Cum Uncis. It has various functions, for instance, warming the kidney yang, nourishing the liver blood, and quenching the liver wind. In the preliminary study, we found that echinacoside, one of the main components of the decoction, exerts remarkable neuroprotective effects on neurodegenerative diseases including PD [10–13]. However, the experimental study on the effect of *Wenshen-Yanggan* decoction on PD has not yet been reported. Therefore, in this study, we aimed to investigate the neuroprotective effects of *Wenshen-Yanggan* decoction on PD model mice and explore its underlying mechanism. The decoction contents were detected, and stable quality control was carried out. Rotenone was used to elicit chronic PD in C57BL/6 mice.

## 2. Materials and Methods

### 2.1. Animals and Groups

10-month-old C57BL/6J male mice weighing 30–35 g were used and bred in the animal facility with a 12 h circadian rhythm (room temperature 22 ± 2°C and humidity 55 ± 5%). Mice were given free access to eat and drink. The animals were acclimated to the environment for 2 weeks before the experiment. Then, they were randomly divided into the blank group (Saline), the model group (RT), the *Wenshen-Yanggan* decoction in high dosage (Wsyg-H), the decoction in medium dosage (Wsyg-M), the decoction in low dosage (Wsyg-L), and the positive control group (Sinemet, carbidopa/levodopa, 25/100 mg. 50 mg/kg), with 10 mice in each group.

### 2.2. Drugs and Reagents

Rotenone (R8875) and sunflower seed oil from *Helianthus annuus* (S5007) were purchased from U S Sigma Company, and Sinemet was purchased from MSD & Co., Inc. The primary antibodies used were as follows: anti-tyrosine hydroxylase antibody (SAB4200697, Sigma), anti-alpha-synuclein antibody (ab59264, Abcam), anti-cleaved caspase-3 antibody (9664s, CST), anti-NF-*κ*B antibody (8242S, CST), anti-phospho-SAPK/JNK antibody (4671, CST), anti-Bcl-2 antibody (12789-1-AP, Proteintech), and anti-GAPDH antibody (60004-1-Ig, Proteintech). The secondary antibodies included HRP-labeled goat anti-rabbit IgG and HRP-labeled goat anti-mouse IgG (ZB-2301 and ZB-2305, Beijing Zhongshan Jinqiao Biotechnology Co., Ltd., China) and goat anti-mouse IgG H&L (Alexa Fluor® 488) (ab150113, Abcam). The ELISA kits were purchased from Shanghai ZCi Biotechnology Co., Ltd., including TNF-*α* (ZC-M6765), IFN-*γ* (ZC-37905), NF-*κ*B (ZC-38232), and IL-1*β* (ZC-37974). Echinacoside (S-003-170119), paeoniflorin (S-010-170214), eugenol lactone (W-022-161216), diosgenin (S-005-170223), and rhynchophylline (G-017-161121) were purchased from Chengdu Ruifensi Biotechnology Co., Ltd. Protocatechuic acid (110809–201205) was purchased from China Food and Drug Administration Research Institute. Cistanches Herba, Paeoniae Radix Alba, Dioscoreae Rhizoma, Linderae Radix, Fructus Alpiniae Oxyphyllae, and Uncariae Ramulus Cum Uncis were purchased from the pharmacy of Nanjing TCM Hospital and authenticated by the Department of Chinese Materia Medica, Nanjing University of Chinese Medicine.

### 2.3. UPLC-MS/MS

Chromatographic conditions: chromatographic column, Agilent ZORBAX Eclipse Plus C18 (2.1 × 50 mm, 1.8 *μ*m), column temperature of 30°C, flow rate of 0.4 mL·min-1, injection volume of 5 *μ*L, and mobile phase: gradient elution of methanol (A)-0.1% aqueous formic acid (B). Gradient elution processes: 0 min, 30% (A); 0.8 min, 90% (A); 1.0 min, 94% (A); 3.5 min, 95% (A); 4.0 min, 95% (A); 4.5 min, 30% (A).

Mass spectrometry conditions: Agilent Company's triple quadrupole mass spectrometer was employed in the experiment. The following conditions were set: electrospray ionization source (ESI), drying gas temperature 350°C, dry gas flow rate 10 L·min^−1^, and capillary voltage 4000 V (+), 3500 V (−). Then, the positive and negative ion scanning mode and the multireaction detection mode (MRM) were selected. The pyrolysis voltage and the detection ions of each component in detection were as follows: echinacoside (175, 785.2^*∗*^/161.0), rhynchophylline (145, 385.2^*∗*^/160.0), chlorhexidine lactone (90, 283.0^*∗*^/265.0), diosgenin (125, 415.2^*∗*^/119.0), protocatechuic acid (85, 153.1^*∗*^/109.0), and paeoniflorin (120, 525.1^*∗*^/449.1), among which, ^*∗*^ refers to the parent ion.

### 2.4. Preparation of the *Wenshen-Yanggan* Decoction

**Figure 1:**
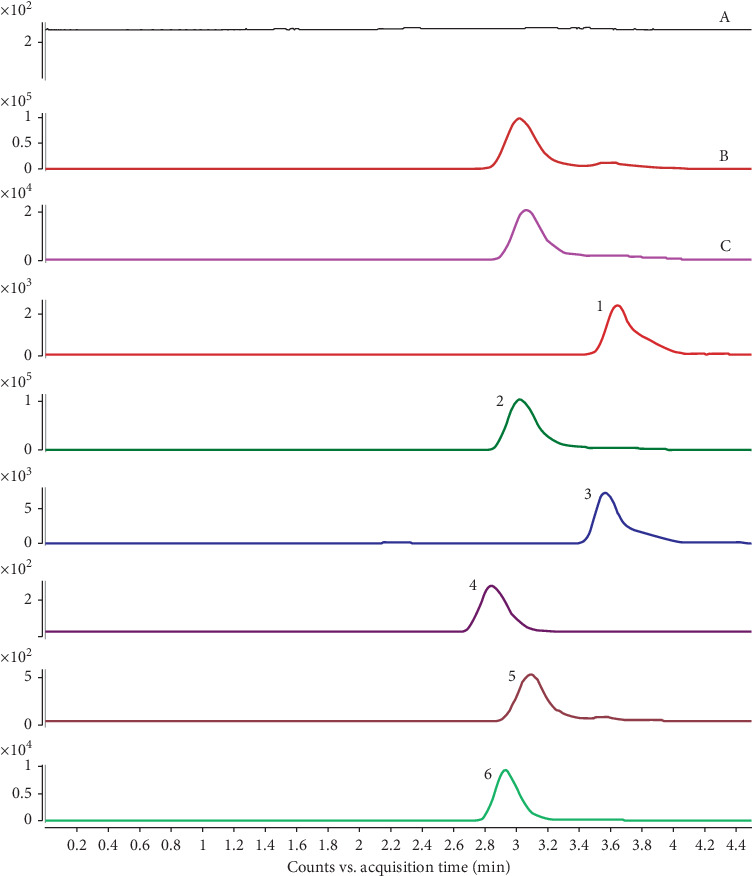
The total ion chromatogram of the *Wenshen-Yanggan* decoction extract. TIC and MRM chromatograms of the sample (A, TIC of the negative sample solution; B, TIC of the mixed reference solution; C, TIC of the sample solution; (1) diosgenin; (2) rhynchophylline; (3) linderane; (4) echinacoside; (5) paeoniflorin; (6) protocatechuic acid).

Linderae Radix (20 g), Fructus Alpiniae Oxyphyllae (30 g), Paeoniae Radix Alba (30 g), and Uncariae Ramulus Cum Uncis (20 g) were mixed together, immersed in 60% alcohol (12 times weight), and decocted for 1.5 h. The extract was filtered through six-layer gauzes. The abovementioned procedures were repeated twice. Residues were collected, and then Dioscoreae Rhizoma (20 g) and Cistanches Herba (30 g) were added, immersed in pure water, and decocted for 1.5 h. This procedure was repeated twice and half volume of the liquid was evaporated. Alcohol was added to the mixture until 50% alcohol content, precipitated it for 48 h, and then recovered and eliminated alcohol until nonalcoholic taste, and the remaining extract is then combined with precipitation solution for concentration until every 1 ml mixture contains 4 g crude drugs. The high-, medium-, and low-dosage solutions of decoction is configured corresponding to 4 g, 2 g, and 1 g crude drugs. The total ion chromatogram and main components of the *Wenshen-Yanggan* decoction extract are displayed in Figure 1 and Table 1.

### 2.5. Modeling and Medication of the Chronic PD Model

The mice in the model group, Wsyg groups, and Sinemet group were intragastrically administrated with rotenone (30 mg/kg) daily for 4 weeks at 10 : 00 am, while the mice in the saline group were treated with equal volume of normal saline. From the 5^th^ week, the mice in the Wsyg groups were treated with low, medium, and high dosage (0.1 ml/10 g) once daily for 4 weeks at 10 : 00 am and the mice in the Sinemet group were treated with Sinemet solution (0.1 ml/10 g). The saline group was treated with equal volume of normal saline. 24 h after the last medication, behavioral tests were carried out. All animals were sacrificed 24 h after the behavioral tests.

### 2.6. Pole Test

According to Chen's method [10], the mouse was placed on the spherical protrusion with its head upward. The time the mouse spent to turn downward (T-turn) and to the bottom of the pole with its posterior limbs touching the ground (T-total) was recorded. Each mouse was tested 3 times at an interval of 2 min, and then the average value was calculated.

### 2.7. Rotarod Test

The rotarod test was performed using a mouse rotarod fatigue meter (ZH-600 Anhui Zhenghua). The mice were placed on a shifting drum (rotation speed: 5 rpm–40 rpm) for 5 minutes 3 times. The time of mice standing on the drum was recorded and averaged.

### 2.8. Immunofluorescence Staining

After the left ventricle perfusion, the brains of mice were rapidly taken and placed in 4% paraformaldehyde overnight. Sucrose solution was used for gradient dehydration, and OCT gel was used for embedding. We prepared the brain tissue sections (30 *μ*m) using a frozen slicer and chose nigral brain slices in line with the brain atlas [14]. After being washed with PBS, goat anti-mouse IgG H&L (1 : 1000) was added and incubated at room temperature in dark for 1 h. After rinsing with PBS, the antifluorescence quenching tablet was added to seal the tablet. An Olympus BX63 fluorescent microscope was used to observe the slices.

### 2.9. Western Blotting

The midbrains of mice were placed in RIPA buffer, the concentration was adjusted evenly, and then they were stored at −70°C. Then, polyacrylamide gel electrophoresis was used. 50 *μ*g of total protein from each sample was separated by SDS-PAGE (10%, 12%, or 15%) and transferred to the polyvinylidene fluoride (PVDF) membrane using the wet transfer approach. Afterward, the PVDF membrane was blocked in 5% BSA for 1h at room temperature and incubated overnight at 4°C with the following primary antibodies: *α*-syn (1 : 1000), TH (1 : 1000), *p*-JNK (1 : 1000), cleaved caspase-3 (1 : 500), Bcl-2 (1 : 1000), NF-*κ*B (1 : 500), and GAPDH (1 : 2000). On the next day, the membrane was rinsed with Tris-HCL buffered salt-Tween (TBST) solution and incubated with the secondary antibody (1 : 2000) for 1h at room temperature; then, the electrochemiluminescent substrate was added. Blots were visualized and analyzed using the Tanon-5200 automatic chemiluminescence image analysis system. The experiment was repeated 3 times.

### 2.10. Enzyme-Linked Immunosorbent Assay (ELISA)

1.5 ml blood of each mouse was collected from the orbit. The blood samples were centrifuged for 10 min at 4°C, 3000 r/min, and 10 cm centrifugal radius. Then, the upper serum was stored at −20°C. The contents of inflammatory factors in the serum of mice including TNF-*α*, IFN-*γ*, IL-1*β*, and NF-*κ*B were detected by using the enzyme-linked immunosorbent assay kit, strictly according to the manufacturer's instructions.

### 2.11. Statistical Processing

All data were expressed as mean ± SEM and analyzed by one-way ANOVA followed by post hoc analysis of the Student–Newman–Keuls test and LSD multiple comparison test. *P* < 0.05 was considered statistically significant.

## 3. Results

### 3.1. Effects of the *Wenshen-Yanggan* Decoction on Behavioral Tests in Rotenone-Induced Chronic PD Model Mice

**Figure 2:**
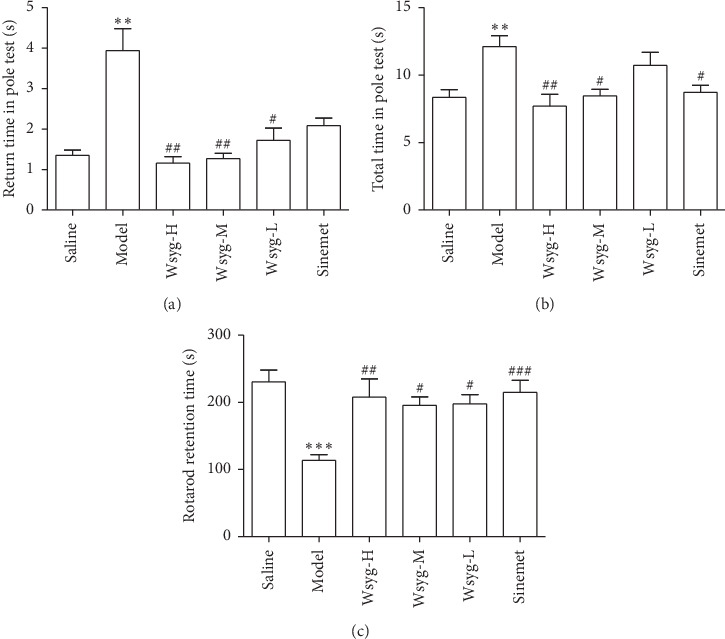
Effects of the *Wenshen-Yanggan* decoction on behavioral tests of the PD chronic model of mice. In the pole test, (a) refers to the turning time and (b) refers to the total time; (c) refers to the retention time in the rotarod test. Saline, blank group; Model, rotenone model group; Wsyg-H, high-dosage group; Wsyg-M, medium-dosage group; Wsyg-L, low-dosage group; Sinemet, positive control. ^*∗∗*^*P* < 0.01 and ^*∗∗∗*^*P* < 0.001 vs. Saline; ^#^*P* < 0.05, ^##^*P* < 0.01, and ^###^*P* < 0.001 vs. Model.

In the pole test, the head-turning time of mice in the rotenone model group (Model) was significantly longer than that in the blank group (Saline) (*P* < 0.01), while it was significantly shortened in the high-dosage (Wsyg-H), medium-dosage (Wsyg-M) and low-dosage group (Wsyg-L) (*P* < 0.01, *P* < 0.01,  and *P* < 0.05). In addition, the mice in Model featured significantly longer total time of climbing than those in Saline (*P* < 0.01), whereas the total time of climbing in Wsyg-H and Wsyg-M groups was significantly decreased (*P* < 0.01 and *P* < 0.05). In the rotarod test, the rod-standing time of Model was significantly shorter than that of Saline (*P* < 0.001); meanwhile, the rod-standing time in Wsyg-H, Wsyg-M, and Wsyg-L groups was significantly longer than that of Model (*P* < 0.01, *P* < 0.05,  and *P* < 0.05) (Figure 2).

### 3.2. Effects of the *Wenshen-Yanggan* Decoction on Midbrain DA Neurons and *α*-Syn in the Rotenone Model Mice

**Figure 3:**
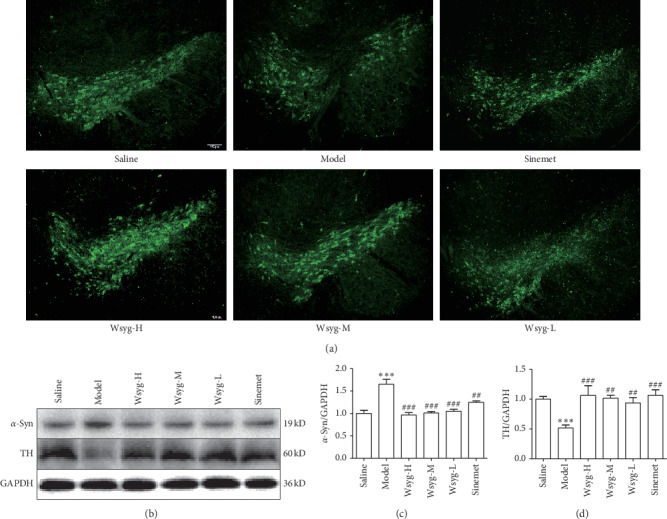
Immunofluorescence staining of TH and *α*-syn protein in the substantia nigra of mice. Saline, blank group; Model, rotenone model group; Wsyg-H, high-dosage group; Wsyg-M, medium-dosage group; Wsyg-L, low-dosage group; Sinemet, positive control. ^*∗∗∗*^*P* < 0.001 vs. Saline; ^##^*P* < 0.01 and ^###^*P* < 0.001 vs. Model.

The number of TH-positive cells reflects the number of DA neurons. The results of western blotting showed that the expression of TH after rotenone modeling was significantly downregulated in comparison with that of Saline (*P* < 0.001), whereas TH expression levels in Wsyg-H, Wsyg-M, and Wsyg-L were profoundly increased relative to Model (*P* < 0.001, *P* < 0.01,  and *P* < 0.01) (Figure 3(b)). Similar results were obtained in the immunofluorescence experiment (Figure 3(a)). Meanwhile, the expression of *α*-syn in the midbrain of mice in Model was significantly elevated compared with Saline (*P* < 0.001). Nonetheless, Wsyg-H, Wsyg-M, and Wsyg-L significantly decreased the expression of *α*-syn (*P* < 0.001, *P* < 0.001, and *P* < 0.001) (Figure 3(b)).

### 3.3. Effects of the *Wenshen-Yanggan* Decoction on Serum Inflammatory Factors including TNF-*α*, IFN-*γ*, IL-1*β*, and NF-*κ*B in Rotenone-Induced PD Model Mice

**Figure 4:**
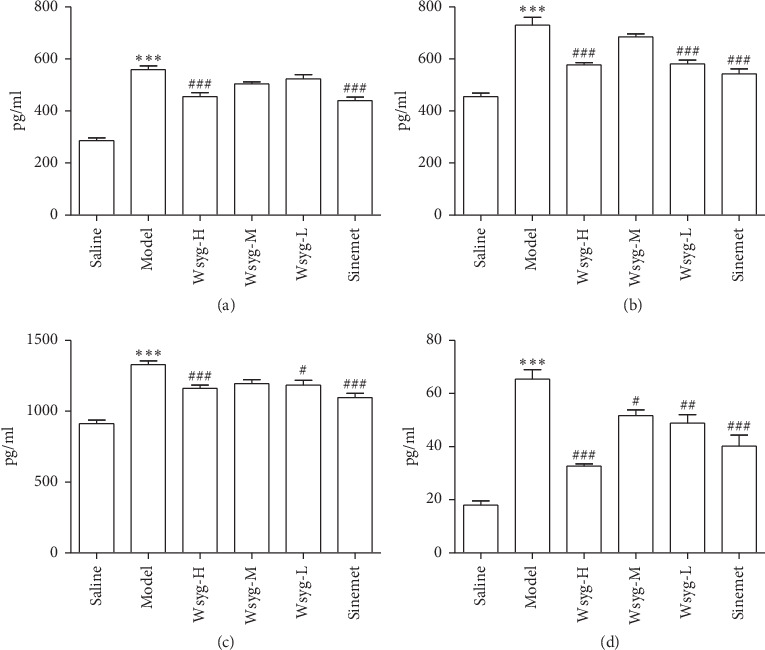
Changes of serum inflammatory factors including (a) TNF-*α*, (b) IFN-*γ*, (c) NF-*κ*B, and (d) IL-1*β* in mice. Saline, blank group; Model, rotenone model group; Wsyg-H, high-dosage group; Wsyg-M, medium-dosage group; Wsyg-L, Low-dosage group; Sinemet, positive control. ^*∗∗∗*^*P* < 0.001 vs. Saline; ^#^*P* < 0.05, ^##^*P* < 0.01, and ^###^*P* < 0.001 vs. Model.

The ELISA showed that the content of inflammatory factors including TNF-*α*, IFN-*γ*, IL-1*β*, and NF-*κ*B in the serum of mice in Model was significantly higher than that in Saline (*P* < 0.001, *P* < 0.001, *P* < 0.001,  and *P* < 0.001), while Wsyg-H can reverse this phenomenon (*P* < 0.001, *P* < 0.001, *P* < 0.001,  and *P* < 0.001). Wsyg-M decreased the content of IL-1*β*(*P* < 0.05), but no significant effect was found on TNF-*α*, IFN-*γ*, and NF-*κ*B. Wsyg-L reduced the content of IFN-*γ*, IL-1*β*, and NF-*κ*B (*P* < 0.001, *P* < 0.05,  and *P* < 0.01), but showed no significant effect on TNF-*α* (Figure 4).

### 3.4. Effects of the *Wenshen-Yanggan* Decoction on the Protein Expression of *p*-JNK, Cleaved Caspase-3, Bcl-2, and NF-*κ*B in the Midbrain of Chronic Rotenone Mice

**Figure 5:**
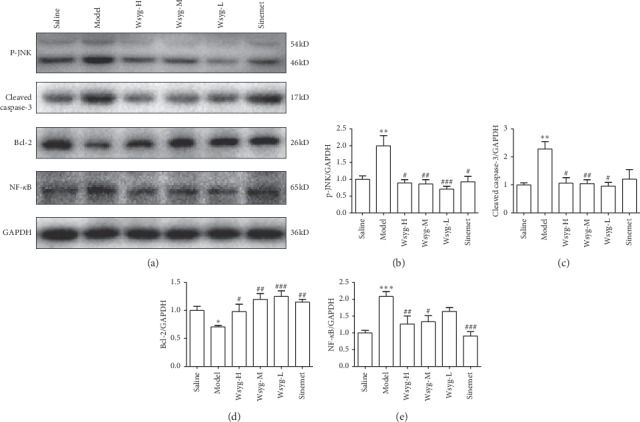
Protein expression of *p*-JNK, cleaved caspase-3, Bcl-2, and NF-*κ*B. Saline, blank group; Model, rotenone model group; Wsyg-H, high-dosage group; Wsyg-M, medium-dosage group; Wsyg-L, low-dosage group; Sinemet, positive control. ^*∗*^*P* < 0.05, ^*∗∗*^*P* < 0.01, and ^*∗∗∗*^*P* < 0.001 vs. Saline; ^#^*P* < 0.05, ^##^*P* < 0.01, and ^###^*P* < 0.001 vs. Model.

Western blotting analysis showed that rotenone induced a marked increase in *p*-JNK, cleaved caspase-3, and NF-*κ*B protein levels in the midbrain (*P* < 0.01, *P* < 0.01,  and *P* < 0.001), while the expression of Bcl-2 was significantly decreased (*P* < 0.05). After treating with high dosage of Wsyg, the expression of *p*-JNK, cleaved caspase-3, and NF-*κ*B was significantly reduced (*P* < 0.05, *P* < 0.05,  and *P* < 0.01), and the level of Bcl-2 was noticeably increased (*P* < 0.05). In addition, *p*-JNK and cleaved caspase-3 and NF-*κ*B protein levels in the midbrain were significantly downregulated (*P* < 0.01, *P* < 0.05,  and *P* < 0.05) in Wsyg-M, while the expression of Bcl-2 was significantly upregulated (*P* < 0.01). The low dosage of the decoction reduced the expression of *p*-JNK and cleaved caspase-3 (*P* < 0.001 and *P* < 0.05) and enhanced the level of Bcl-2 (*P* < 0.001), but did not affect NF-*κ*B Figure 5.

## 4. Discussion

PD is a slowly progressive neurodegenerative disorder. The pathological changes of PD are characterized by the presence of SNpc degeneration and the occurrence of Lewy body, which are related to autophagy, apoptosis, oxidative stress, and other complex mechanisms including immune response, inflammatory response, and excitotoxicity [15]. The focus of PD research studies is to study its etiology and develop therapies that possess disease-modifying effect and are able to slow down the disease process.

Although the pathogenesis of PD is still unclear, it is believed that neuroinflammation is one of the main pathological mechanisms of PD and a potential target for PD treatment [16]. Upon autopsy of animal models and PD patients, the expression of proinflammatory cytokines in cerebrospinal fluid (TNF-*α*, IL-1*β*, IFN-*γ*, and so on) was increased in PD [17]. In this study, the same phenomenon occurred in the rotenone model group, while the *Wenshen-Yanggan* decoction could reduce these inflammatory factors in PD mice. NF-*κ*B, an important nuclear transcription factor, plays a significant role in cell growth and proliferation and is involved in the etiology and anti-inflammatory effects of PD. The inflammatory response can activate NF-*κ*B, leading to increased synthesis of the proinflammatory factor TNF-*α* protein [18]. The *Wenshen-Yanggan* decoction reduced the level of NF-*κ*B in both the serum and midbrain, which might partially account for *Wenshen-Yanggan* decoction-generated inhibition of inflammatory response in PD mice.

Neuronal apoptosis, specifically the apoptosis of dopaminergic neurons in the dense substantia nigra, has been reported to be the final outcome of PD. [19]. Similarly, we observed the upregulation of cleaved caspase-3 in the brain of the PD model mice in this experiment. The activation of caspase-3 is related to the apoptosis of neurons. The JNK signaling pathway is involved in the regulation of cell growth, differentiation, apoptosis, and so on, and its abnormality is closely related to PD-related apoptosis in the substantia nigra [5, 20, 21].

Caspase-3 is a main apoptosis-related enzyme, which is capable of activating the caspase cascade. The activation of JNK signaling pathway can upregulate the expression of COX-2 and caspase-3. Once JNK is activated, *p*-JNK stimulates the Bcl-2/Bax family and its downstream substrates, promoting apoptosis. [22] Bcl-2 is a membrane-bound antiapoptotic protein regulated by the upstream JNK, [23] which can attenuate the antiapoptotic effects after activation [24].

In conclusion, this study showed that (1) the *Wenshen-Yanggan* decoction could improve the PD-like behaviors in the rotenone-induced PD model mice, reduce the expression level of *α*-syn, and prevent the loss of substantia nigra DA neurons; (2) the rotenone-induced PD model mice displayed neuroinflammatory symptoms including increased expression of inflammatory factors in the serum and midbrain, which were improved by the *Wenshen-Yanggan* decoction; (3) the *Wenshen-Yanggan* decoction restored the elevated expression of the key apoptotic protein, cleaved caspase-3, antiapoptotic protein, Bcl-2 protein, and the apoptotic pathway-related protein, *p*-JNK, in rotenone-induced PD model mice, suggesting that the *Wenshen-Yanggan* decoction might suppress apoptosis in PD mice through JNK controlled inhibition of inflammatory response. This experiment has proven that the *Wenshen-Yanggan* decoction could exert protective effects in PD nerve injury and illustrated its potential mechanism. The present study has shed light on the development of traditional Chinese medicine decoction for PD treatment.

## Figures and Tables

**Table 1:** Identification of the main components of the *Wenshen-Yanggan* decoction.

Component	Regression equation (*μ*g/ml)	Linear range	*R* ^2^	Content (*μ*g/g)
Echinacoside	*y* = 136.6601*x* − 93.5573	1.1975–66.2651	0.9991	57.6387
Paeoniflorin	*y* = 337.0665*x* + 121.5458	0.4770–65.7875	0.9989	181.5809
Linderane	*y* = 3482.2138*x* + 4052.7251	0.0047–66.4502	0.9981	3.1658
Protocatechuic acid (PCA)	*y* = 6940.1857*x* − 8744.4088	1.5984–66.3660	0.9999	10.3859
Diosgenin	*y* = 175.3360*x* + 268.6082	4.9431–660.2135	0.9986	123.4398
Rhynchophylline	*y* = 98336.3056*x* + 136871.6067	1.7286–66.5304	0.9999	25.5002

## Data Availability

The data used to support the findings of this study are available from the corresponding author upon request.

## References

[1] Wang S., Yuan Y.-H., Chen N.-H., Wang H.-B. (2019). The mechanisms of NLRP3 inflammasome/pyroptosis activation and their role in Parkinson’s disease. *International Immunopharmacology*.

[2] Booth H. D. E., Hirst W. D., Wade-Martins R. (2017). The role of astrocyte dysfunction in Parkinson’s disease pathogenesis. *Trends in Neurosciences*.

[3] Gelders G., Baekelandt V., Van der Perren A. (2018). Linking neuroinflammation and neurodegeneration in Parkinson’s disease. *Journal of Immunology Research*.

[4] Trompetero A., Gordillo A., Del Pilar M. C., Cristina V. M., Bustos Cruz R. H. (2018). Alzheimer’s disease and Parkinson’s disease: a review of current treatment adopting a nanotechnology approach. *Current Pharmaceutical Design*.

[5] Bohush A., Niewiadomska G., Filipek A. (2018). Role of mitogen activated protein kinase signaling in Parkinson’s disease. *International Journal of Molecular Sciences*.

[6] Zhang Z.-N., Zhang J.-S., Xiang J. (2017). Subcutaneous rotenone rat model of Parkinson’s disease: dose exploration study. *Brain Research*.

[7] Maniyath S. P., Solaiappan N., Rathinasamy M. (2017). Neurobehavioural changes in a hemiparkinsonian rat model induced by rotenone. *Journal of Clinical and Diagnostic Research: JCDR*.

[8] Subaraja M., Vanisree A. J. (2016). Rotenone causing dysfunctional mitochondria and lysosomes in cerebral ganglions of Lumbricus terrestris degenerate giant fibers and neuromuscular junctions. *Chemosphere*.

[9] Xue X., Bian J.-S. (2015). Neuroprotective effects of hydrogen sulfide in Parkinson’s disease animal models. *Methods in Enzymology*.

[10] Chen C., Xia B., Tang L. (2019). Echinacoside protects against MPTP/MPP+ induced neurotoxicity via regulating autophagy pathway mediated by Sirt1. *Metabolic Brain Disease*.

[11] Liang Y., Chen C., Xia B. (2019). Neuroprotective effect of echinacoside in subacute mouse model of Parkinson’s disease. *BioMed Research International*.

[12] Zhang Y., Long H., Zhou F. (2017). Echinacoside’s nigrostriatal dopaminergic protection against 6-OHDA-induced endoplasmic reticulum stress through reducing the accumulation of Seipin. *Journal of Cellular and Molecular Medicine*.

[13] Shiao Y.-J., Su M.-H., Lin H.-C., Wu C.-R. (2017). Echinacoside ameliorates the memory impairment and cholinergic deficit induced by amyloid beta peptides via the inhibition of amyloid deposition and toxicology. *Food & Function*.

[14] Paxinos G., Franklin K. B. J. (2004). *The Mouse Brain in Stereotaxic Coordinates*.

[15] Pang S. Y., Ho P. W., Liu H. F. (2019). The interplay of aging, genetics and environmental factors in the pathogenesis of Parkinson’s disease. *Translational Neurodegeneration*.

[16] Russo I., Bubacco L., Greggio E. (2014). LRRK2 and neuroinflammation: partners in crime in Parkinson’s disease?. *Journal of Neuroinflammation*.

[17] De Lella Ezcurra A. L., Chertoff M., Ferrari C., Graciarena M., Pitossi F. (2010). Chronic expression of low levels of tumor necrosis factor-*α* in the substantia nigra elicits progressive neurodegeneration, delayed motor symptoms and microglia/macrophage activation. *Neurobiology of Disease*.

[18] Kaltschmidt C., Kaltschmidt B., Neumann H., Wekerle H., Baeuerle P. A. (1994). Constitutive NF-kappa *B* activity in neurons. *Molecular and Cellular Biology*.

[19] Mori T., Hayashi T., Su T.-P. (2012). Compromising *σ*-1 receptors at the endoplasmic reticulum render cytotoxicity to physiologically relevant concentrations of dopamine in a nuclear factor-*κ*B/Bcl-2-dependent mechanism: potential relevance to Parkinson’s disease. *Journal of Pharmacology and Experimental Therapeutics*.

[20] He C., Zhu H., Li H., Zou M.-H., Xie Z. (2013). Dissociation of Bcl-2-Beclin1 complex by activated AMPK enhances cardiac autophagy and protects against cardiomyocyte apoptosis in diabetes. *Diabetes*.

[21] Hosseinzadeh L., Monaghash H., Ahmadi F., Ghiasvand N., Shokoohinia Y. (2017). Bioassay-guided isolation of neuroprotective fatty acids from nigella sativa against 1-methyl-4-phenylpyridinium-induced neurotoxicity. *Pharmacognosy Magazine*.

[22] Lei K., Davis R. J. (2003). JNK phosphorylation of Bim-related members of the Bcl2 family induces Bax-dependent apoptosis. *Proceedings of the National Academy of Sciences*.

[23] Zhong L., Shu W., Dai W., Gao B., Xiong S. (2017). Reactive oxygen species-mediated *c*-Jun NH_2_-terminal kinase activation contributes to hepatitis *B* virus *X* protein-induced autophagy via regulation of the beclin-1/Bcl-2 interaction. *Journal of Virology*.

[24] Bassik M. C., Scorrano L., Oakes S. A., Pozzan T., Korsmeyer S. J. (2004). Phosphorylation of BCL-2 regulates ER Ca^2+^ homeostasis and apoptosis. *The EMBO Journal*.

